# Investigation of Oxidative DNA Damage Levels in Urine of Healthcare Workers Exposed to Ionizing Radiation

**DOI:** 10.3390/toxics13110990

**Published:** 2025-11-17

**Authors:** Ayşegül Yurt, Ayşe Coşkun Beyan, Gamze Tuna, Yaşar Aysun Manisalıgil, Sabriye Özcan, Hande Oğuzhan

**Affiliations:** 1 Institute of Health Sciences, Department of Medical Physics, Dokuz Eylul University, Izmir 35340, Türkiye; 2 Vocational School of Health Science, Medical Imaging Techniques, Dokuz Eylul University, Izmir 35340, Türkiye; aysun.manisaligil@deu.edu.tr; 3 Department of Occupational Medicine, Faculty of Medicine, Dokuz Eylul University, Izmir 35340, Türkiye; ayse.coskun@deu.edu.tr; 4 Institute of Health Sciences, Department of Molecular Medicine, Dokuz Eylul University, Izmir 35340, Türkiye; tungamze@gmail.com (G.T.);; 5 Medical Laboratory Techniques Program, Vocational School of Health Services, Dokuz Eylul University, Izmir 35340, Türkiye; 6 BioIzmir-Izmir Health Technologies Development and Accelerator Research and Application Center, Dokuz Eylul University, Izmir 35340, Türkiye; 7 Department of Occupational Health and Safety, Dokuz Eylul University, Izmir 35340, Türkiye; sabriyekaratas.ozcan@gmail.com

**Keywords:** oxidative stress, radiation dose, radiation effect, work health, risk assessment

## Abstract

This study aimed to assess oxidative DNA damage products in healthcare workers who are directly exposed to or use ionizing radiation in their work. In the study, three groups were defined based on the probability of radiation exposure, ranging from the highest-risk group to the lowest, with the fourth group designated as the control group. First, a questionnaire was administered to the participants, and then their first morning urine samples were taken to detect oxidative DNA damage markers. According to the Kruskal-Wallis test results among the four groups in our study, statistically significant differences were found only in terms of age, height, and weight (*p* values = 0.011, 0.038, and 0.003, respectively). However, it was observed that there was no significant relationship between the oxidative DNA damage parameters 8-hydroxy-2′-deoxyguanosine (8-OH-dG), and 8,5′-cyclo-2′-deoxyadenosines (S- and R-cdA) in relation to radiation exposure, with *p*-values of 0.132, 0.179, and 0.611, respectively. The study’s results revealed that exposure to ionizing radiation did not cause a significant increase in urinary oxidative DNA damage markers. This outcome may be associated with the effective use of personal protective equipment and strict adherence to radiation safety protocols among healthcare workers.

## 1. Introduction

After the discovery of X-rays in 1895 and the subsequent understanding of radioactivity, the biological effects of ionizing radiation became well established [1]. While extensive research has focused on the outcomes of high ionizing radiation doses, a growing body of studies is investigating the effects of low doses [1,2,3]. While the consequences of high doses have been documented through catastrophic events like the Chernobyl disaster and the Hiroshima bomb, the long-term effects of low doses remain a subject of ongoing research [4,5]. Assessing health effects related to radiation exposure poses a significant challenge in establishing cause-and-effect relationships, particularly concerning potential long-term effects, such as genotoxicity, within the Occupational Health and Safety (OHS) framework [6]. At the cellular level, ionizing radiation causes DNA damage, which can lead to uncontrolled cell division, cell death, and the production of reactive oxygen species, also known as free radicals [7,8]. Indirectly, ionizing radiation triggers the formation of reactive oxygen species (ROS) through the hydrolysis of water. These ROS can inflict damage on nucleic acids, proteins, and lipids. Notably, DNA damage gives rise to 2′-deoxynucleoside lesions, such as 8-hydroxy-2′-deoxyguanosine (8-OH-dG) and the S and R forms of 8,5′-cyclo-2′-deoxyadenosine (S-cdA, R-cdA). DNA repair enzymes excise these damaged products, which are then excreted from the body via urine [9]. Numerous studies have investigated oxidative stress in healthcare workers (HCWs) exposed to radiation [8,9,10,11], as well as the oxidant/antioxidant capacities of these workers [8,12,13,14,15]. These studies highlight the reality of radiation-induced oxidative stress in individuals and emphasize the importance of interventions, such as antioxidant therapy, to mitigate its effects. Furthermore, research suggests that stable end products of lipid peroxidation resulting from oxidative stress, such as 4-hydroxynonenal (4-HNE) and 8-OH-dG, can influence DNA methylation [14,15]. The biological effects of ionizing radiation encompass both early and late manifestations, including the generation of reactive oxygen species (ROS), apoptosis, and DNA damage such as double-strand breaks, chromosomal abnormalities, micronuclei formation, and nucleoplasmic bridges (NPBs) formation, as well as the upregulation of stress-responsive genes [7,8,10,14]. Thus, comprehending these potential biological effects is particularly important for HCWs categorized by radiation risk. Therefore, in the realm of OHS, it is imperative to determine whether biological effects exist and, if so, their magnitude among HCWs operating in radiation-exposed areas classified by levels of risk. Considering all these studies, numerous factors influencing oxidative stress have been identified, highlighting the significance of investigating 8-OH-dG, S-cdA, and R-cdA as markers of DNA damage in the morning urine of radiation-exposed healthcare workers [14]. Despite an understanding of various factors affecting oxidative stress in this population, assessing DNA damage markers, specifically 8-OH-dG, S-cdA, and R-cdA levels in initial urine samples, remains crucial [14,15]. This study aimed to investigate the relationship between radiation exposure and oxidative DNA damage, focusing on parameters such as 8-OH-dG, S-cdA, and R-cdA, in HCWs who are exposed or likely to be exposed to ionizing radiation.

## 2. Materials and Methods

### 2.1. Study Groups, Inclusion, and Exclusion Criteria, and Radiation Exposure Risk Assessments

The minimum sample size required to investigate oxidative DNA damage within each group was calculated to be 26 workers per group, ensuring a 95% confidence level and an 80% power. Consequently, each group comprised 25 to 30 healthcare workers. The study by Viegas et al. served as a reference for the sample size calculation [16], and all groups consisted of 30 participants each. Although the sampling method was not random, all individuals registered with our hospital’s occupational health unit and those who volunteered to participate were included in the study. Exclusion criteria included pregnancy, a cancer diagnosis within the last two years, and severe infection within the past week.

Ionizing radiation exposure was evaluated both qualitatively and quantitatively using multiple complementary methods. 

### 2.2. Occupational Health and Safety Unit (OHSU) Risk Assessment

Risk assessments of HCWs’ radiation exposure were categorized as high, medium, or low based on evaluations conducted by the Hospital Occupational Health and Safety Unit (OHSU), using the 5 × 5 matrix method (Supplementary Data), expert opinions, and a literature review [17,18]. Annual dosimetry results of employees were obtained from the institution’s radiation safety committee. Accordingly, the annual average whole-body dose values were as follows: Group 1 (0.645 ± 1.89 mSv), Group 2 (0.068 ± 0.08 mSv), and Group 3 (0.034 ± 0.05 mSv). None of the dosimeter readings of our participants exceeded the nationally or internationally accepted annual radiation dose limits. Strict regulations regarding the use of PPE are enforced by the hospital administration and unit supervisors, in accordance with national regulations [19].

### 2.3. Workers’ Perception of Radiation Risk

The radiation risk perception of the workers was measured with the question, “How much radiation exposure do you think you have while working?” The answers were grouped as “none,” “I am exposed to a little,” “I am exposed to medium,” and “I am exposed to a lot.” The dosimeter results of the HCWs.

### 2.4. National Legal Regulation on Radiation Regulation

According to the national regulation on radiation, “controlled” and “monitored” areas have been decided [19]. Groups have been identified based on qualitative and quantitative data of radiation exposure. Group 1 includes HCWs with the highest probability of exposure due to the patient becoming a radiation source when radionuclides are administered; HCWs face a high risk due to scattered radiation in coronary and interventional angiography applications. HCWs in Group 2 are shielded by walls and doors between the control desk and the CT and mammography patient scanning rooms; therefore, the risk for those working in this area is less than that of the other groups. Group 3 was defined as a group with a lower probability of radiation exposure than the previous groups. It includes those who are not typically radiation workers but work in environments where radiation is used as part of their job duties. Group 4 consisted of individuals working in supervised areas who were not radiation workers. Qualitative and quantitative data determining the radiation exposure risk categories of the groups are presented in Table 1.

### 2.5. Data Collection

Each participant signed an informed consent form and then completed the questionnaire form through face-to-face interviews. These included socio-demographic information (4 questions), a history of chronic diseases, regular medication use, overall health status (7 questions), smoking and alcohol consumption habits (2 questions), and information on their work life and environment and self-assessment of individual risk factors (8 questions).

### 2.6. Chemicals and Standards

Oasis HLB Extraction Cartridges (reversed phase with 60 mg sorbent weight) were procured from Waters Corp. (Milford, MA, USA). Syringe filters with a pore size of 0.22 µm were sourced from Labsolute (Geyer GmbH & Co., Renningen, Germany). Tubes with a molecular mass cut-off of 3000 Da were obtained from Pall (Pall Corporation, Port Washington, NY, USA). Alkaline phosphatase (ALP), formic acid, and acetonitrile were acquired from Merck (Darmstadt, Germany). The internal standards R-cdA-^15^N_5_, S-cdA-^15^N_5_, and 8-OH-dG-^15^N_5_ were supplied by the National Institute of Standards and Technology (NIST, Gaithersburg, MD, USA).

### 2.7. Collection of Biological Samples and Measurement of Oxidative DNA Damage Parameters

The assessment of damaged DNA nucleosides via liquid chromatography-tandem mass spectrometry (LC-MS/MS) was conducted at the Research Laboratory of the Department of Medical Biochemistry at the University. Morning first urine samples, totaling 5 mL, were collected from healthcare workers for analysis. All urine samples were aliquoted after collection and stored at −80 °C until further analyses.

To quantify oxidative DNA damage, LC-MS/MS with stable-isotope dilution (SID) was employed using multiple reaction monitoring (MRM) acquisition modes, following a previously published protocol [20]. Internal standards containing ^15^N_5_-labeled analogs of the analytes were added to each urine sample before the extraction step. A mixture of internal standards, consisting of 46.8 µL of 8-OH-dG-^15^N_5_ (0.002 mmol/L), S-cdA-^15^N_5_ (0.021 μmol/L), and R-cdA-^15^N_5_ (0.042 μmol/L), was added to 1 mL of the urine samples [21]. The use of these stable isotope–labeled compounds follows the isotope dilution mass spectrometry approach described by Dizdaroglu and co-workers [22], ensuring accurate quantification and correction for sample-to-sample variations. The isotope-labeled standards serve to normalize for possible losses during extraction, enzymatic hydrolysis, and instrumental analysis, thereby improving precision and comparability among all samples. Subsequently, the samples were centrifuged at 1000× *g* for 15 min, followed by filtration using nylon syringe filters. The filtered samples were loaded onto extraction cartridges, washed with 2 mL of distilled water, and eluted with 1 mL of 30% methanol. Eluted samples were then dried using a vacuum concentrator (Thermo Scientific SpeedVac, Marietta, OH, USA) and reconstituted in 100 µL of digestion buffer (1 mol/L sodium acetate, 10 mmol/L Tris-HCl, pH 7.5). Hydrolysis with alkaline phosphatase (22 units per sample) was performed at 37 °C for 1 h, followed by filtration using three kDa tubes via centrifugation at 5000× *g* for 50 min.

Analysis was performed using high-performance liquid chromatography (HPLC) coupled with a mass spectrometer equipped with a triple quadrupole ion trap (4000 QTRAP, Applied Biosystems, Foster City, CA, USA), operating in positive ionization mode. Separation was achieved using an LC column (2.1 mm × 150 mm, 3.5 µm particle size; Zorbax SB-Aq column, Agilent Technologies, Santa Clara, CA, USA) with an attached C8 guard column (2.1 mm × 12.5 mm, 5 µm particle size). Mobile phases consisted of water containing 0.1% formic acid (mobile phase A) and acetonitrile containing 0.1% formic acid (mobile phase B). Analysis by LC–MS/MS with MRM was performed using the mass/charge (*m*/*z*) transitions *m*/*z* 284, *m*/*z* 168, and *m*/*z* 289, *m*/*z* 173 for 8-OH-dG and 8-OH-dG-^15^N_5_, respectively, and with *m*/*z* transitions *m*/*z* 250, *m*/*z* 164, and *m*/*z* 255, *m*/*z* 169 for the S- and R-diastereomers of cdA and cdA-^15^N_5_, respectively. Results were normalized using urinary creatinine concentrations, with values expressed in nmol/mmol creatinine.

### 2.8. Statistical Analyses

Data were analyzed using SPSS (Statistical Package for the Social Sciences) version 29.0 software. In descriptive analyses, frequency data were presented as numbers (n) and percentages (%), and the Chi-square (χ^2^) test was employed for comparing categorical variables. The normal distribution of continuous variables was assessed using the Kolmogorov-Smirnov and Shapiro-Wilk tests. For variables not conforming to a normal distribution, the Mann-Whitney U test and the Kruskal-Wallis analysis was conducted to compare two groups and three or more groups, respectively. Subsequently, following the Kruskal-Wallis analysis, Dunn-Bonferroni post hoc tests were applied to identify the group or groups responsible for significant differences (SPSS, 2024). The correlation between continuous variables and DNA damage parameters was evaluated using the Spearman correlation. Statistical significance was set at *p* < 0.05 for all tests.

## 3. Results

The study examined oxidative DNA damage parameters in urine, as well as demographic traits such as age, biological sex, smoking habits, and chronic diseases, in groups of HCWs, as presented in Table 2. The mean age of Group 2 is 33.43 ± 10.53 years, while the other groups were 40.10 ± 10.48 years (Group 1), 40.50 ± 9.27 years (Group 3), and 43.60 ± 9.17 years (Group 4), respectively. Group 2 was statistically significantly younger (*p* = 0.011).

Significant differences were observed in height (*p* = 0.038) and weight (*p* = 0.003) between groups. Accordingly, Bonferroni-adjusted post hoc analyses were performed. Group 2 had a significantly lower mean age and mean weight compared to the other groups. The proportion of females was highest in Group 4 (80%) and lowest in Group 3 (45.8%). However, no statistically significant difference in biological sex distribution was found among the groups (*p* = 0.081). There were no significant differences among the groups in terms of alcohol consumption (three or more days per week), smoking status, or the presence of chronic diseases, as well (Table 2).

The mean duration of employment was 9.10 ± 8.62 years in Group 1, 10.41 ± 11.49 years in Group 2, and 13.50 ± 10.10 years in Group 3. No significant differences were observed among the groups (*p* = 0.249). Night shift proportions were similar in Groups 1, 2, and 3 (all above 30%), with none of the Group 4 participants reporting night shifts (*p* = 0.014). This suggests that there is complexity in the relationship between the groups, highlighting the need for further research to better understand and resolve any potential discrepancies. All participants in Groups 1 and 2 used personal dosimeters, which distinguished them from the other groups, and this distinction was statistically significant (*p* < 0.001). The proportion of participants who responded “very high exposure” to the question “How much radiation are you exposed to in your working environment?” was 51.7% in Group 1, 13.0% in Group 2, and 29.2% in Group 3. Group 1 reported significantly higher perceived radiation exposure compared to the other groups (*p* = 0.021) (Table 3).

**Figure 1:**
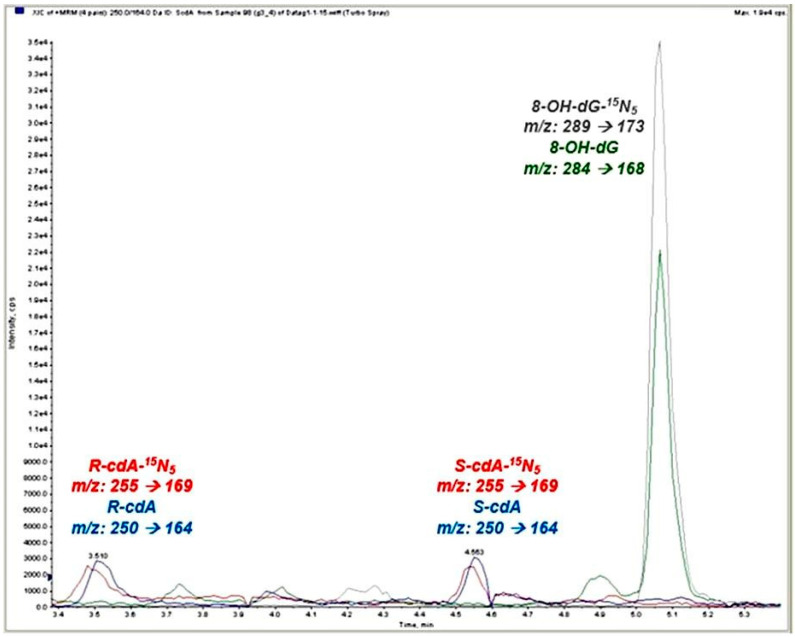
Ion-current profiles of the 8-OH-dG, 8-OH-dG-^15^N_5_, R-cdA, S-cdA, R-cdA-^15^N_5_, and S-cdA-^15^N_5_ mass transitions. Colored chromatographic peaks represent the corresponding analytes and their isotope-labeled internal standards: green, 8-OH-dG; gray, 8-OH-dG^15^N_5_; blue, R/S-cdA; red, R/S-cdA^15^N_5_.

**Figure 2:**
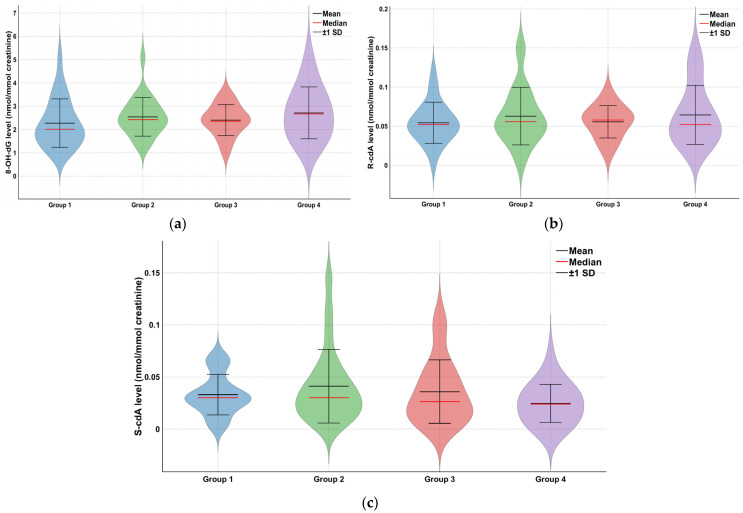
Changes in oxidative stress parameters of Groups 1, 2, 3, and the control group. (**a**) 8-OH-dG, (**b**) R-cdA, (**c**) S-cdA.

The analysis of oxidative DNA damage parameters involved determining the levels of 8-OH-dG, S-cdA, and R-cdA. Figure 1 showcases representative ion-current profiles of the mass transitions *m*/*z* 284 → 168 (8-OH-dG), *m*/*z* 289 → 173 8-OH-dG-^15^N_5_, *m*/*z* 250 → 164 (R- and S-cdA), and *m*/*z* 255 → 169 (R- and S-cdA-^15^N_5_), captured during the LC-MS/MS analysis of urine samples. Changes in mean and standard deviation values of oxidative stress parameters such as 8-OH-dG, R-cdA, and S-cdA determined by the Kruskal-Wallis test for all groups were described with violin graphs (Figure 2).

The mean 8-OH-dG level was 2.27 ± 1.04 in Group 1, 2.54 ± 0.83 in Group 2, 2.41 ± 0.67 in Group 3, and 2.71 ± 1.11 in Group 4. No statistically significant differences were observed among the groups (*p* = 0.132). A similar pattern was observed for S-cdA and R-cdA levels. No significant differences were found among the groups (*p* = 0.179 and *p* = 0.611, respectively) (Table 4). Since our data were not normally distributed, Spearman’s correlation was applied, and the results are presented in the Supplementary Materials. According to the table, no significant correlation was observed between 8-OH-dG, S-cdA, and R-cdA levels and the workers’ age, weekly working hours, or total years of employment (*p* > 0.05) (see Supplementary Materials Table S2).

## 4. Discussion

In our study, no significant differences were observed in DNA damage parameters among the risk groups established based on both qualitative and quantitative assessments of radiation exposure. The absence of an association between the measured/estimated risk and the observed biological effects suggests that additional factors should be considered. In the literature, studies frequently report increased DNA damage among workers employed in areas known or measured to pose a high radiation risk [8,9,10,11,16]. However, the lack of elevated DNA damage in workers from high-risk areas may serve as evidence that protective measures play an important role in mitigating biological effects. The use of personal protective equipment, such as lead aprons and thyroid shields, by healthcare workers in the work environment, combined with the presence of a ventilation system, minimizes radiation exposure and ensures that it remains below the acceptable dose limits defined in national and international regulations and standards.

In a similar study conducted among healthcare workers at Dokuz Eylül University, where we investigated genotoxicity, we observed that during the COVID-19 period, administrative measures such as reduced working hours, lower patient load, and rotational shifts led to a significant decrease in previously elevated occupational hygiene measurements. These two studies together illustrate how occupational hygiene measures—particularly administrative controls and the consistent use of personal protective equipment—can effectively reduce, if not completely eliminate, occupational risks associated with ionizing radiation exposure [23].

Although markers such as γH2AX foci, dicentric chromosomes, micronuclei, and chromosomal translocations are recognized as highly specific indicators of ionizing radiation exposure, their applicability in population-based or occupational settings is often limited by methodological complexity, higher cost, and the requirement for viable cells or fresh samples. In contrast, oxidative DNA damage markers such as 8-OH-dG and R/S-cdA offer several practical advantages. These lesions are formed as a direct consequence of hydroxyl radical attack on DNA and represent a stable and quantifiable footprint of oxidative stress, which is one of the primary pathways through which low-dose ionizing radiation exerts its biological effects. Radiation workers are exposed to secondary X-rays and gamma rays and therefore receive low radiation doses with low Linear Energy Transfer (LET).

The measurement of oxidative DNA damage products in non-invasive samples such as urine provides a sensitive and ethical means to assess the cumulative biological impact of chronic low-dose exposure. Numerous studies have demonstrated the value of 8-OH-dG as an early biomarker of oxidative stress in healthcare workers and other populations exposed to radiation [24,25,26]. While these oxidative lesions may not be exclusively specific to ionizing radiation, they are mechanistically relevant to radiation-induced ROS generation and therefore constitute an integral part of the biological response to radiation exposure. In this context, the findings of the present study provide complementary insight into oxidative stress-mediated DNA damage rather than direct strand break events. The integration of oxidative DNA damage assessment with radiation-specific endpoints, such as γH2AX or micronucleus frequency, in future studies could further enhance the mechanistic understanding of chronic low-dose radiation exposure in healthcare professionals.

Chen et al. examined Dnmts, 8-OH-dG, and 4-HNE parameters to assess oxidative DNA damage associated with ionizing radiation exposure during interventional procedures conducted by physicians [14]. Radiation exposure during interventional operations impacted the expression of enzymes involved in DNA methylation, which in turn affected DNA methylation processes. Additionally, it was highlighted that oxidative damage induced by ionizing radiation influences DNA methylation. This study, consistent with our findings, also found no correlation between radiation exposure. We did not analyze confounders such as age and gender that may affect DNA damage, nor were there any significant differences between the groups.

Among the commonly used approaches in radiation research, antioxidant enzyme activities and lipid peroxidation metabolites are frequently measured as indicators of oxidative stress response rather than direct quantification of DNA damage [9,11,12,14,15,27]. In these studies, oxidative DNA damage and antioxidant parameters in HCWs exposed to ionizing radiation have been explored, analyzing markers such as malondialdehyde (MDA), superoxide dismutase (SOD), catalase, and reduced glutathione in blood samples [1]. These investigations revealed elevated MDA levels alongside significantly reduced SOD levels. Other studies focused on lipid peroxidation levels, specific concentrations of biomarkers, and total antioxidant capacity in blood as indicators of oxidative stress induced by ionizing radiation. These studies showed increased lipid peroxidation and decreased antioxidant capacity, reflecting higher levels of ROS. The results of these studies vary considerably. The lack of standardization and validation of these methods, along with their high susceptibility to confounding factors, remains a significant limitation [9,11,12,14,15,27].

The effects of subacute/chronic low-dose exposure on DNA repair proteins have sparked new discussions. While the annual dose in our study remained below regulatory limits (20 mSv on average over the 5-year period for radiation workers), the literature on low-dose ionizing radiation (LDIR) (commonly defined as low dose (<100 mGy) or a low dose rate (<0.1 mGy/min) of ionizing radiation) suggests that even chronic low-dose exposures can activate cellular stress responses and modulate DNA repair-related signaling pathways. Previous studies have reported adaptive responses characterized by altered antioxidant defenses, DNA repair signaling, and reduced damage formation following subsequent exposures (e.g., dose-rate effects and radiation hormesis phenomena). These effects have been described primarily in controlled in-vitro or animal models, and data in occupational settings remain limited and heterogeneous [28,29,30].

A significant limitation of our study is its cross-sectional design, which may affect the precision of results. Long-term monitoring of genotoxicity parameters could offer more accurate insights, as demonstrated in previous examples [26]. Additionally, our study only investigated two genotoxicity parameters, while the literature includes studies that use various methods as discussed in detail. However, in order to conduct a cost-effective study, we preferred to use a method that was already validated and available within our institution’s infrastructure. Furthermore, volunteer selection may have biased the sample, since workers who were more aware of the issue were likely to participate (bias against the null hypothesis). An essential limitation of the study is that radiation risks affecting oxidative DNA damage cannot be measured individually due to the coexistence of multiple risks in the work environment. Besides previous work history, other factors that were not measured or determined may have influenced oxidative stress markers and genotoxicity. Individual differences in DNA repair capacity, dietary antioxidant intake, and underlying health conditions may also impact the results. Although the sample size was determined according to the minimum required calculation, larger studies are needed to enable more comprehensive analyses. Some subgroup analyses were not performed due to the limited sample size. Overcoming these limitations and conducting large-scale studies would require financial support, likely from national authorities.

## 5. Conclusions

In our study, all exposed groups used personal dosimeters and PPE, with higher PPE use in the highest-risk units; cumulative doses remained well below regulatory limits. Our finding of urinary oxidative DNA damage is therefore consistent with effective radiation-protection practices at our institution; however, the cross-sectional design precludes causal inference, so we refrain from attributing these results specifically to training or monitoring. Furthermore, considering prior reports on adaptive cellular responses to low-dose radiation, including modulation of DNA damage signaling and repair pathways, these results are compatible with, though do not directly demonstrate, subtle biological adaptation mechanisms.

The results of this study can be considered preliminary. Analyzing these data from routine health screenings over an expanded sample space and by year will help obtain more precise and reliable results. Since the analysis methods used to determine oxidative DNA damage markers in the study have high costs, it is necessary to ensure the continuity of the study with a larger-budget project and discuss data regarding changes over the years.

## Figures and Tables

**Table 1:** Identification of groups according to risks of radiation exposure.

Groups	Risk Category	Annual Average Whole-Body Dose Values (Mean + SD)	OHS Unit 5 × 5 Matrix Risk Assessment Results	Self-Assessment Thoughts on Radiation Exposure (%)	
Group 1 (n = 29)	High-risk HCWs (angiography, interventional radiology, nuclear medicine)	(0.645 ± 1.89 mSv)	Purple It is considered a “controlled area” according to national legislation [19].	High Moderate Low None	15 (51.7%) 7 (24.1%) 7 (24.1%) -
Group 2 (n = 23)	Moderate-risk HCWs (CT, mammography)	(0.068 ± 0.08 mSv)	Red It is considered a “monitored area” according to national legislation [19].	High Moderate Low None	3 (13.0%) 14 (60.9%) 6 (26.1%) -
Group 3 (n = 24)	Non-radiology HCWs with possible exposure (orthopedics, neurosurgery, OR staff, endoscopy)	(0.034 ± 0.05 mSv)	Green and Yellow It is considered a monitored area according to national legislation [19].	High Moderate Low None	7 (29.2%) 6 (25.0%) 10 (41.7%) 1 (4.2%)
Group 4 (n = 20)	Control group—no radiation exposure (other hospital units)	-	None	High Moderate Low None	- - 5 (25.0%) 15 (75.0%)

**Table 2:** The characteristics of the groups.

Characteristics	Exposed Groups (%)			Group 4 (Control Group)	*p* *
	Group 1	Group 2	Group 3		
Age Mean ± SD (min–max)	40.10 ± 10.48 (24–58)	33.43 ± 10.53 (22–59)	40.50 ± 9.27 (26–60)	43.60 ± 9.17 (32–60)	0.011
Height (cm) Mean ± SD (min–max)	168.97 ± 10.26 (152–194)	164.04 ± 8.37 (150–180)	171.54 ± 8.79 (154–185)	166.30 ± 8.23 (150–183)	0.038
Weight (kg) Mean ± SD (min–max)	72.86 ± 13.68 (45–98)	61.96 ± 13.76 (43–101)	76.92 ± 6.78 (47–120)	67.85 ± 1.37 (48–90)	0.003
Biological Sex (%) Female Male	62.1 37.9	73.9 26.1	45.8 54.2	80.0 20.0	0.081
Smoking Status ** Non-smokers Smokers	79.3 20.7	65.2 34.8	75.0 25.0	85.0 15.0	0.710
Alcohol consumption more than 3 times a week (Yes) (%)	3.4	0.0	0.0	5.0	0.730
Chronic disease (Yes) (%)	31.0	8.7	41.7	35.0	0.077
Regular exercise (Yes) (%)	6.9	4.3	16.7	4.3	0.240

* Kruskal-Wallis, Mean-standard deviation (Mean ± SD). ** Smoking status was defined as follows: Yes: Patients who are currently active smokers or have a history of smoking/No: Patients who have never smoked.

**Table 3:** Working life parameters and risk perception of the groups.

	Group 1 Mean ± SD (Min–Max)		Group 2 Mean ± SD (Min–Max)		Group 3 Mean ± SD (Min–Max)		Group 4 Mean ± SD (Min–Max)		* *p*
Weekly work hours (h)	36.79 ± 3.23 (35–45)		35.74 ± 7.94 (5–48)		45.21 ± 7.90 (35–70)		40.75 ± 1.83 (40–45)		<0.001
Working year (year)	9.10 ± 8.62 (1–31)		10.41 ± 11.49 (0–40)		13.50 ± 10.10 (0–34)		11.80 ± 9.93 (1–34)		0.249
Regularly Using (PPE) **	27 *** (93.1%)		19 *** (82.6%)		19 *** (79.1%)		-		
Personal Dosimeter Usage Yes (%)	100.0		100.0		83.3		0.0		<0.001
Night Shift Yes (%)	31.0		34.8		41.7		0.0		0.014
Self-assessment thoughts on radiation exposure n (%)	High	15 (51.7%)	High	3 (13.0%)	High	7 (29.2%)	High	-	
	Moderate	7 (4.1%)	Moderate	14 (60.9%)	Moderate	6 (25.0%)	Moderate	-	0.021
	Low	7 (24.1%)	Low	6 (26.1%)	Low	10 (41.7%)	Low	5 (25.0%)	
	None	-	None	-	None	1 (4.2%)	None	15 (75.0%)	

* Kruskal-Wallis, Mean-standard deviation (Mean ± SD). ** In our country, national regulations regarding radiation work require radiation workers to wear goggles, thyroid protection, gonadal protection, and a lead apron. In rare cases, one or more of these PPE items may be worn. *** Missing data.

**Table 4:** Oxidative stress parameters of the radiation workers and the control group.

Group	8-OH-dG (nmol/mmol Creatinine) Mean ± SD (Min–Max)	S-cdA (nmol/mmol Creatinine) Mean ± SD (Min–Max)	R-cdA (nmol/mmol Creatinine) Mean ± SD (Min–Max)
Group 1 (n = 29)	2.27 ± 1.04 (0.90–5.16)	0.03 ± 0.02 (0.0005–0.07)	0.05 ± 0.03 (0.00003–0.12)
Group 2 (n = 23)	2.54 ± 0.83 (1.38–5.10)	0.04 ± 0.04 (0.003–0.14)	0.06 ± 0.04 (0.0014–0.15)
Group 3 (n = 24)	2.41 ± 0.67 (0.98–3.58)	0.04 ± 0.03 (0.001–0.11)	0.06 ± 0.02 (0.013–0.09)
Group 4 (n = 20)	2.71 ± 1.11 (1.13–5.20)	0.02 ± 0.02 (0.002–0.07)	0.06 ± 0.04 (0.025–0.14)
* *p* value	0.132	0.179	0.611

* Kruskal-Wallis, Mean-standard deviation (Mean ± SD).

## Data Availability

The datasets generated during and/or analyzed during the current study are not publicly available but may be obtained from the corresponding author upon reasonable requests in accordance with personal data protection laws.

## Supplementary Materials

The following supporting information can be downloaded at: https://www.mdpi.com/article/10.3390/toxics13110990/s1, Table S1: 5 * 5 risk assessment of the occupational health and safety unit at Dokuz Eylul University Hospital; Table S2: Correlation analysis of oxidative stress parameters.

## Abbreviations

The following abbreviations are used in this manuscript:

HCWs	Healthcare workers
ILO	International Labour Organization
WHO	World Health Organization
ROS	Reactive Oxygen Species
GDP	Gross Domestic Product
OHS	Occupational Health and Safety
OHSU	Hospital Occupational Health and Safety Unit
CT	Computed Tomography
ALP	Alkaline Phosphatase
LC-MS/MS	Liquid Chromatography-Tandem Mass Spectrometry
HPLC	High-Performance Liquid Chromatography
PPE	Personal Protective Equipment
MDA	Malondialdehyde
SOD	Superoxide Dismutase
