# Supplementary material for: Investigation of Oxidative DNA Damage Levels in Urine of Healthcare Workers Exposed to Ionizing Radiation
Source: Toxics. 2025 Nov 17;13(11):990. doi: 10.3390/toxics13110990 (PMC12655978; doi:10.3390/toxics13110990)
Supplement: Supplementary file 1 [file toxics-13-00990-s001.zip › toxics-3939996-supplementary.pdf]

Supplementary Table S1. 5*5 risk assessment of the occupational health and safety unit at Dokuz Eylul University Hospital

COLOR	POINT	DESCRIPTION
Green	0.1 - 1.5	The factor is in the environment but does not generate risks.
Blue	1.6 - 6.5	The risk factor is present and under control, or it does not result in irreparable or persistent consequences.
Yellow	6.6 - 12.5	The factor could lead to health problems or adverse outcomes. It might generate problems during control.
Red	12.6 – 20.5	The factor could lead to serious and urgent health issues. Precaution is absolutely important.
Purple	20.6 – 25.0	The control of the fact matters vitally; consequences could be lethal. It is not possible to work on that risk level.

Supplementary Table S2. Correlation analysis of oxidative stress parameters

Spearman Correlation	Age		Weekly work hours		Working year	
	**Correlation coefficient**	**p**	**Correlation coefficient**	**p**	**Correlation coefficient**	**p**
**(8-OH-dG)**	0,099	0,337	0,006	0,951	0,101	0,329
**S-cdA**	0,122	0,237	0,009	0,927	0,033	0,753
**R-cdA**	0,151	0,142	0,166	0,106	0,181	0,080
